# Novel Insight Into the Development and Function of Hypopharyngeal Glands in Honey Bees

**DOI:** 10.3389/fphys.2020.615830

**Published:** 2021-01-22

**Authors:** Saboor Ahmad, Shahmshad Ahmed Khan, Khalid Ali Khan, Jianke Li

**Affiliations:** ^1^Key Laboratory of Pollinating Insect Biology, Institute of Apicultural Research, Ministry of Agriculture, Chinese Academy of Agricultural Sciences, Beijing, China; ^2^Laboratory of Apiculture, Department of Entomology, Pir Mehr Ali Shah (PMAS)- Arid Agriculture University, Rawalpindi, Pakistan; ^3^Research Center for Advanced Materials Science (RCAMS), King Khalid University, Abha, Saudi Arabia; ^4^Unit of Bee Research and Honey Production, Biology Department, Faculty of Science, King Khalid University, Abha, Saudi Arabia

**Keywords:** exocrine glands, nurse bees, acini, proteomics, protein and gene expression, royal jelly, enzyme

## Abstract

Hypopharyngeal glands (HGs) are the most important organ of hymenopterans which play critical roles for the insect physiology. In honey bees, HGs are paired structures located bilaterally in the head, in front of the brain between compound eyes. Each gland is composed of thousands of secretory units connecting to secretory duct in worker bees. To better understand the recent progress made in understanding the structure and function of these glands, we here review the ontogeny of HGs, and the factors affecting the morphology, physiology, and molecular basis of the functionality of the glands. We also review the morphogenesis of HGs in the pupal and adult stages, and the secretory role of the glands across the ages for the first time. Furthermore, recent transcriptome, proteome, and phosphoproteome analyses have elucidated the potential mechanisms driving the HGs development and functionality. This adds a comprehensive novel knowledge of the development and physiology of HGs in honey bees over time, which may be helpful for future research investigations.

## Introduction

Exocrine glands have the potential to release their secretions to the external of the insect through a duct and can be categorized into pheromone producing (mandibular glands) and non-pheromone producing (hypopharyngeal glands) structures. The hypopharyngeal glands (HGs) were first reported in 1846 by Nickel (Cruz-Landim and Costa, 1998), and are only present in hymenopteran insects. However, HGs morphologically and physiologically vary across and within the eusocial insect species (Cruz-Landim and Costa, 1998; Britto et al., 2004; Amaral and Caetano, 2005).

Insect glands perform various functions including communication, diet processing, reproduction, defense, and nest construction (Chapman et al., 2013). In eusocial insects, HGs have been studied in honey bees (Wang and Moeller, 1971; Knecht and Kaatz, 1990; Yousef et al., 2014), stingless bees (Cruz-Landim et al., 1986; Britto et al., 2004), wasps (Cruz-Landim and Costa, 1998), and ants (Gama, 1985). In honey bees, HGs are well-developed in worker bees as compared to queens and drones and degenerate when the tasks switch from nursing in the hive to foraging in the field (Robinson, 1992; Britto et al., 2004). The HGs are critical in secreting the royal jelly proteins, that play essential roles in the diet and caste differentiation of honey bees (Kamakura, 2011). Some stingless bees (Meliponini) possess HGs in female castes while in other species both female and male have HGs (Costa and Cruz-Landim, 1999). In wasps, the HGs have no anatomical variations among the castes and play a vital role in nest building and production of food for larvae (Cruz-Landim and Costa, 1998; Britto et al., 2004). In ants, the HGs reach their highest development in queen, are small in the worker and even smaller in male (Gama, 1985). Their function in the ants may aid in brood care (Wilson, 1980) and enzyme production that helps in the digestion of food (Ayre, 1967; Gama, 1985).

The histological and ultrastructural details as well as the biochemical functions of the HGs of various species of honey bees have been studied (Deseyn and Billen, 2005; Suwannapong et al., 2007, 2010; Kheyri et al., 2012; Richter et al., 2016). Researchers are seeking ways to increase the production of royal jelly (RJ) by the HGs and to determine its chemical composition (Nie et al., 2017; Altaye et al., 2019a; Al-Kahtani and Taha, 2020). RJ is a natural valuable bee product and has a wide range of applications in cosmetics, medicine, and dietary supplements for promotion of human health (Ramadan and Al-Ghamdi, 2012; Ahmad et al., 2020). Here, we summarize the morphogenesis and functions of HGs in the pupae and through the adult life of worker bees, to begin to gain a comprehensive mechanistic understanding of their development and physiology, also highlights the knowledge gap for the future investigations.

## Morphogenesis of HGs in Honey Bees

HGs display morphological plasticity and changes during the developmental stages of honey bees (Huang and Robinson, 1996; Ohashi et al., 2000; Deseyn and Billen, 2005; Liu et al., 2014; Richter et al., 2016). The fine structure of HGs start to develop in pupae about a week before they would have emerged as adults and continue to change structure until they die (Painter and Biesele, 1966). In the adult bees, HGs are well-developed but their development and maturation during metamorphosis is still unknown. The detail morphogenesis of HGs during pupal and adult stages of honey bees are described below.

### Pupal Stage

**Figure 1:**
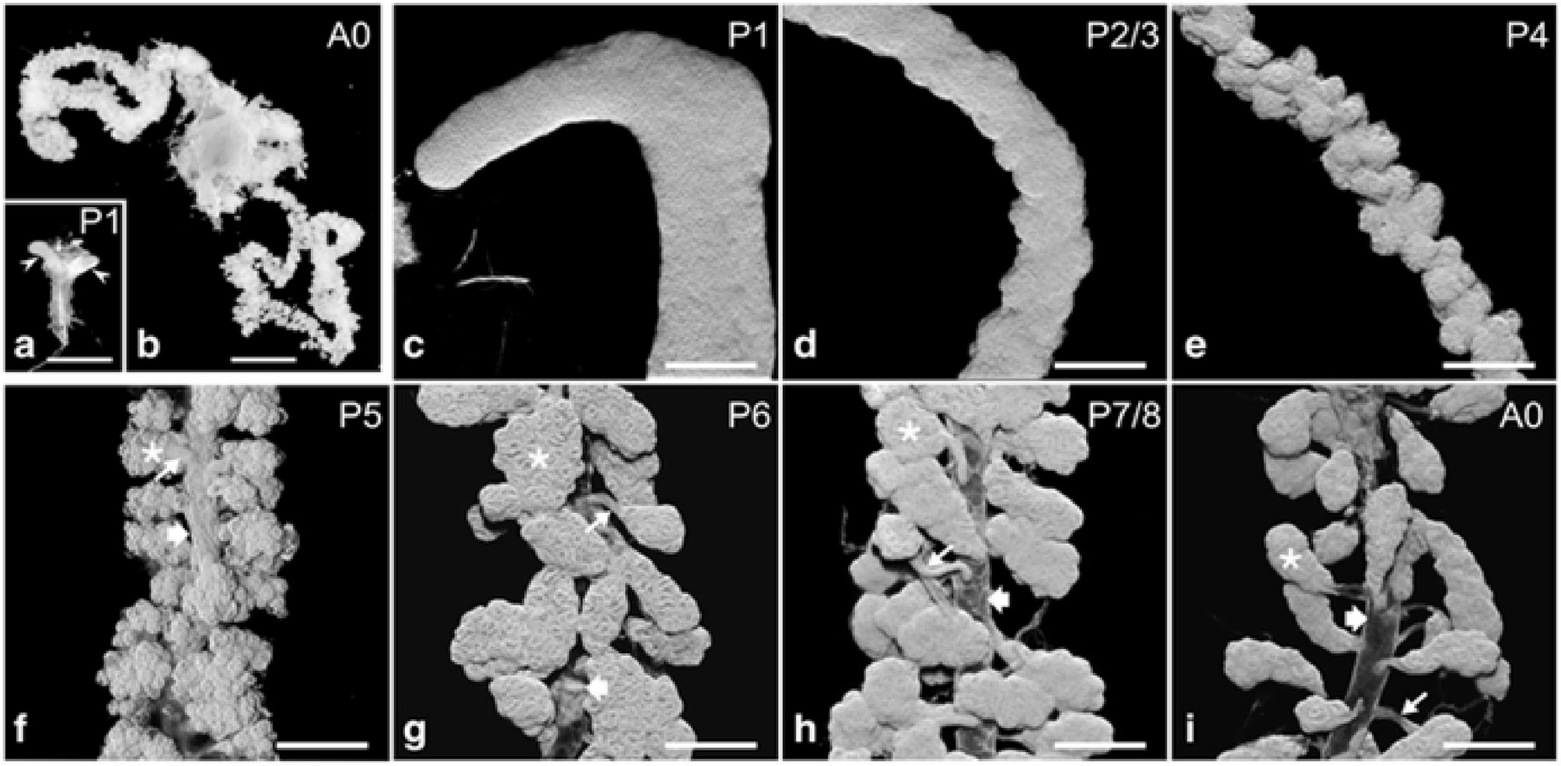
Hypopharyngeal gland (HG) morphogenesis. **(a)** Macroscopic images of HG primordia (arrowheads) at pupal stage P1. [**(b)**; A0] HGs in a new emerged bee (NEB). **(c–i)** HGs during pupal development (P1–P7/P8) and of a NEB (A0). Asterisks, acini; thin arrows, bundle of ductules; broad arrows, collecting duct. Data were used from Klose et al. (2017).

During the pupal stages (from P1 to P9), the development process of HGs could be divided into several key events (Figure 1) (Groh and Rössler, 2008; Klose et al., 2017). At pupal stage P1, two saccule-like evaginations, representative of HGs primordia, extend from the lower side of the pharynx. Also, the saccule comprises of transparent epithelium infoldings with a large lumen and a smooth outer surface about 0.5 and 0.2 mm in length and width, respectively. At this stage mitotic nuclei are noted in the apical, middle, and basal portion of epithelium (Painter and Biesele, 1966; Klose et al., 2017).

Furthermore, the basal portion has nuclei that contain condensed and fragmented chromatin (Škerl and Gregorc, 2015; Klose et al., 2017). Therefore, a wide range of protein molecules have to be produced to enhance cell proliferation, tissue development, and a large amount of biological fuels are engaged to maintain this protein synthesis (Feng et al., 2009; Li et al., 2010; Liu et al., 2014; Qi et al., 2015). Also, the amino acids, ribosomes and proteasomes are produced as the HGs develop (Li et al., 2010; Liu et al., 2014). At the development stage P2/3, the thickness of epithelium remains uniform and three types of cells could be separated by their differences in morphology and position. Two cells are recognized as the secretory and the duct cells, and the third cell is termed the accessory cell (Painter, 1945; Klose et al., 2017). Five to 15 sets of three-cell units (secretory, duct, and accessory cells) are organized in clusters next to each other within the epithelium. One cluster of cells differentiates into the acinus, canal bundle, and collecting duct epithelium. At pupal stage P4, the HG primordium enlarges in length and decreases its width, and the outer surface becomes undulating. At P5 stage, three-cell units form a gland that is arranged into hundreds of cauliflower structures, representing future acini connected by canal cells to a collecting duct (Klose et al., 2017). During the P6–P8 development stage, three cell-units become two-cell units through the apoptotic removal of the accessory cells. At this stage, the HGs have adopted the morphology of adult glands, with the several transparent acini of oval shape linked by the canal cell to the collecting duct which extends along the entire length of glands (Škerl and Gregorc, 2015; Klose et al., 2017).

#### Development of Canaliculus and F-actin Ring in the Pupal Stage

**Figure 2:**
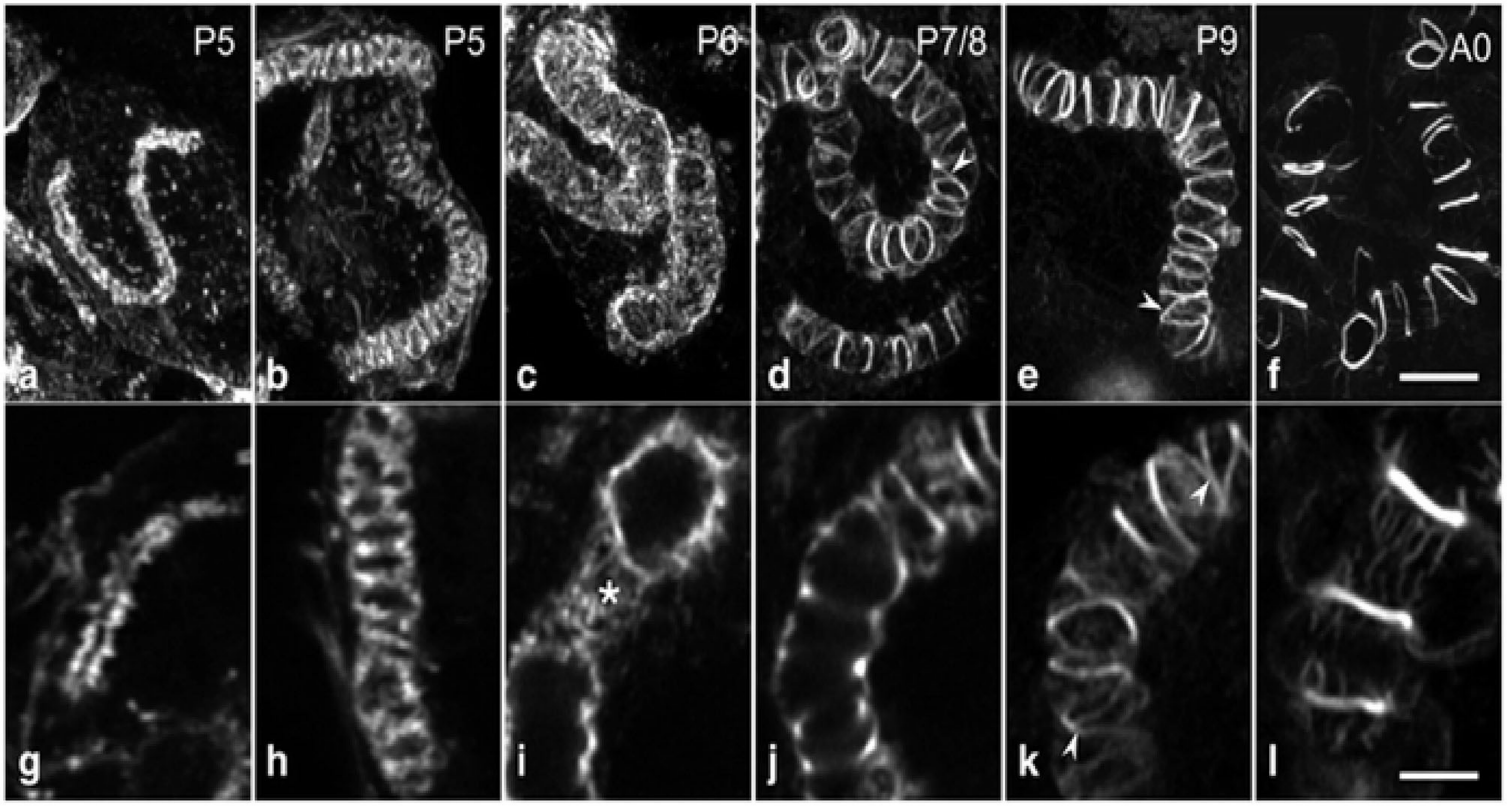
A systematic morphogenesis of the canaliculus in secretory cells during the second half of pupal development. **(a–f)** Maximum intensity projections of image stacks. **(g–l)** Individual optical sections at a higher magnification. At P5 **(a,g)**, the F-actin tube, representing F-actin associated with the developing canaliculus is thin and short with a narrow lumen. **(b,h)** The F-actin tube has enlarged in length and diameter. At P6 **(c,i)**, a dense web of F-actin (asterisk) forms an expanded tube of uniform diameter. Between P7 and exclusion of the worker bees **(d–f,j–l)**, F-actin in the tube becomes reorganized and concentrated into rings. Ring distance increases during developmental progression, whereas the number of interconnections (arrowheads) and the amount of F-actin in association with the inter-ring segments decrease. Data were taken from Klose et al. (2017).

From cell-biological perspective, the canaliculus is a blind-ending lumen in the secretory cells of HGs that is enclosed by the apical membrane (Beams and King, 1933; Beams et al., 1959; Painter and Biesele, 1966; Richter et al., 2016). Furthermore, a series of visible actin rings surround the end apparatus inside the secretory cells, which are essential for holding the end apparatus in place as the secretion swells the extracellular partitions between the end apparatus and the cell membrane (Kheyri et al., 2012). At the pupal stage, the exit timing, and of the creation of canaliculus of the secretory cells are still unknown, indicating that these cells lack a canaliculus through pupal maturation. During stages P2–P4, the F-actin structure is seen in the interior of the secretory cells, and the canaliculus has not yet formed (Klose et al., 2017). In P5 secretory cells, a single tube-like F-actin extends from the bottom terminal of the ductule into the secretory cell for some distance. Moreover, the F-actin structures are associated with the internal membranes, and tubes of increasing length are seen in the consecutive development stages of canaliculus formation (Klose et al., 2017). At stage P6, the canaliculus radially expands to a diameter over its whole length without any periodic constrictions (Klose et al., 2017). By stage P7/P8, the canaliculus-associated F-actin structure is concentrated and frequently interconnected. The space between the actin rings is covered with a sparse matrix of actin filaments (Klose et al., 2017). During the subsequent pupal developmental stage, the actin rings become much obvious, the amount of interconnection decreases and the space between the adjacent actin rings expands (Figure 2). Furthermore, the F-actin ring portion decreases during the last few days of pupal development (Klose et al., 2017).

**Figure 3:**
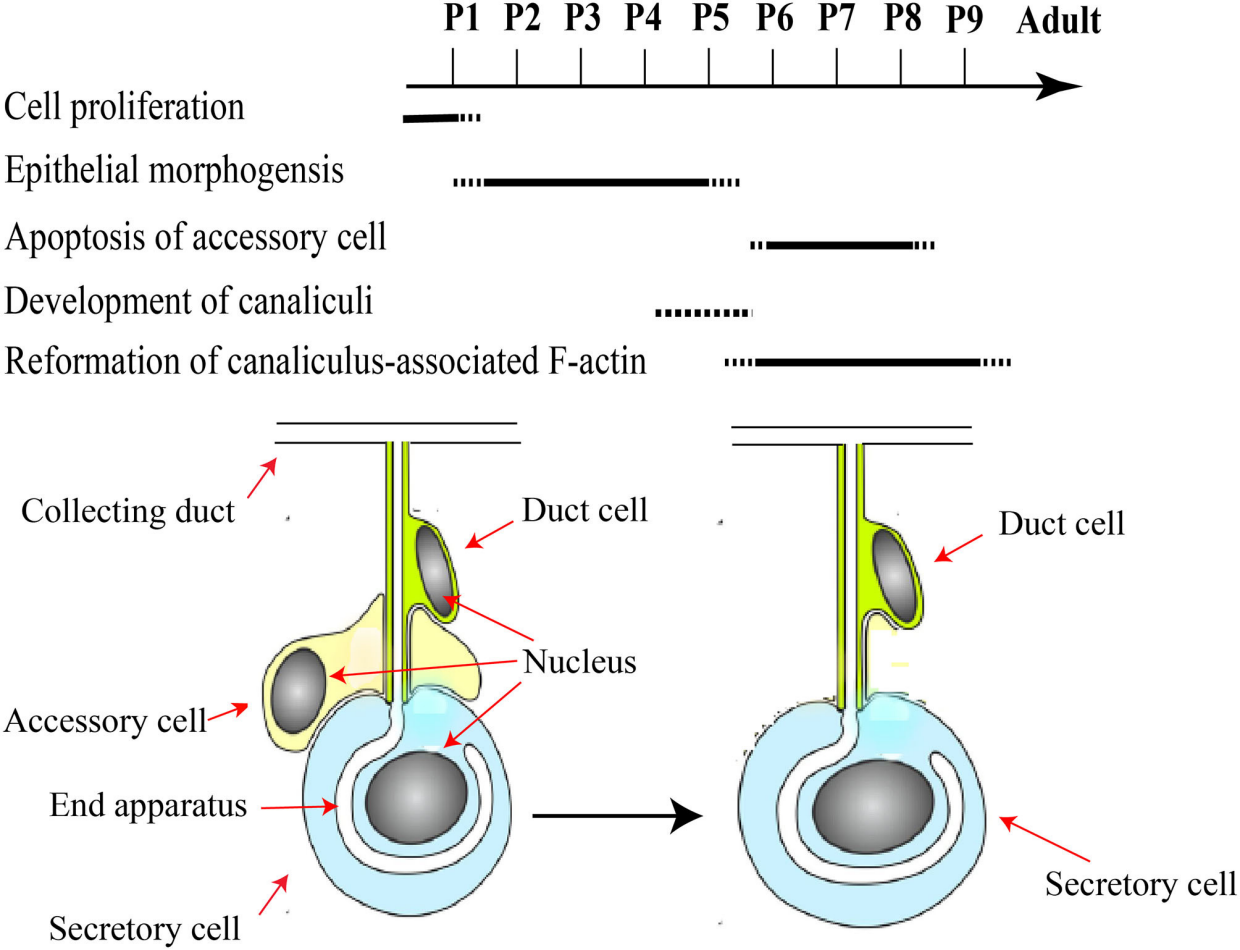
A schematic framework of key events during pupal development of HGs. Major developmental pupal stages P1–P9 assigned according to the literature. Pupal HGs composed of three-cell units. Accessory cells are removed from the three-cell units to acquire the final units of a secretory cell and duct cell. Modified from Groh and Rössler (2008), Klose et al. (2017), and (Richter et al., 2016).

In short, HGs in the pupal stage develop as follows (Figure 3): cell proliferation occurs in a pseudostratified epithelium and forms three-cell units within the epithelial layer (Painter, 1945; Groh and Rössler, 2008; Klose et al., 2017). Accessory cells are removed from the three-cell units to acquire the final units of a secretory cell and duct cell, and the canaliculus is an invagination and expansion of the apical membrane, which has at its end a related actin cytoskeleton that form unique actin ring (Beams et al., 1959; Richter et al., 2016; Klose et al., 2017). Moreover, the HGs are composed of immature secretory units that are inconsistent in shape and contain 8–10 secretory cells (Suwannapong et al., 2010).

### HGs Development Across the Adult Worker Bees

**Figure 4:**
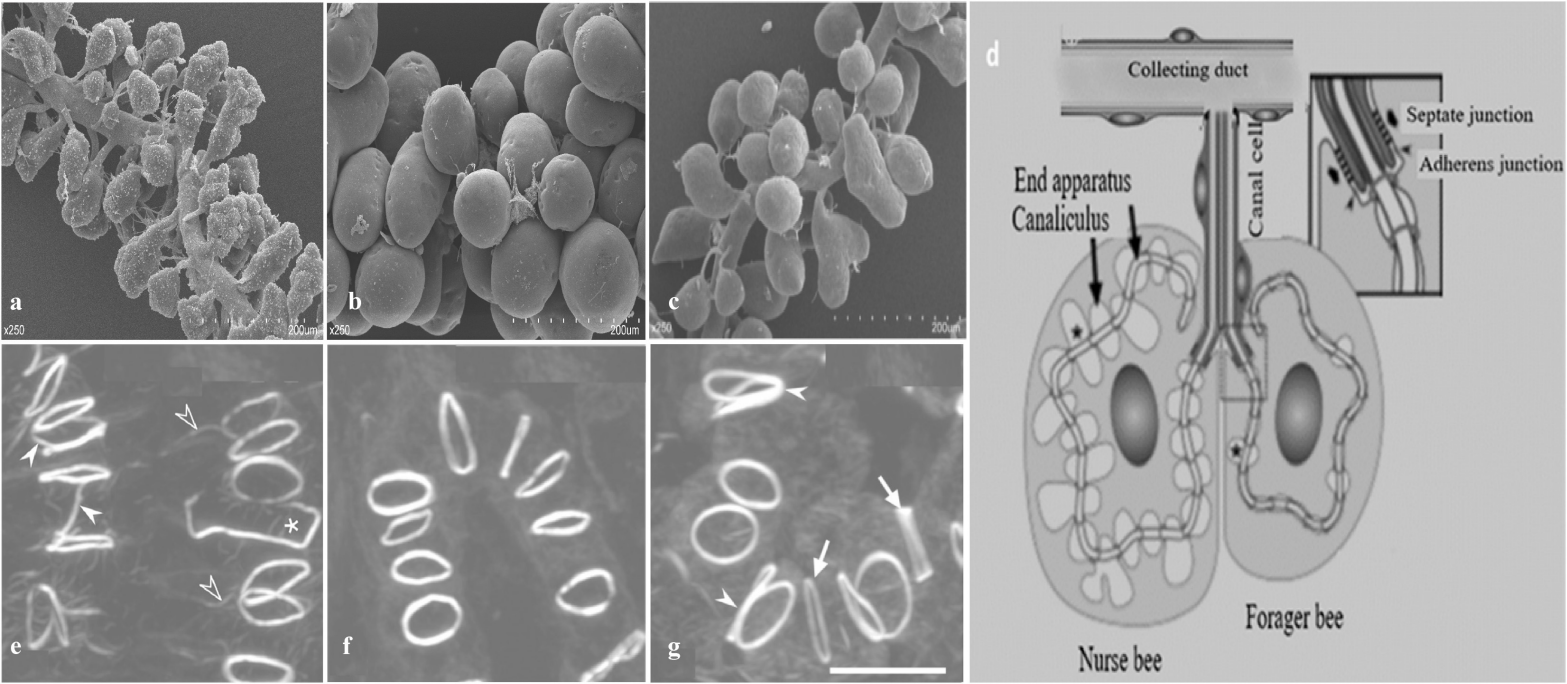
A schematic diagram of hypopharyngeal gland (HG) development in worker honey bees. **(a)** In newly emerged bees (NEBs) **(b)** well developed in nurse bees (NBs) **(c)** shrink during forager bees (FBs) **(d)** Schematic arrangement of an acinus. Each acinus is consisted of several secretory cells and attached via a junctional complex (arrowheads) to a canal cell that delivers secretory products to the collecting duct. Formation of actin rings **(e)** In NEBs, some actin rings have F-actin rays (open arrowheads), are interconnected with each other (white arrowheads), or are open (asterisk) **(f)** actin rings are well developed in NBs **(g)** FBs have larger actin rings of varying thickness (arrows) and are sometimes in contact (white arrowheads). Modified from Qi et al. (2015) and Richter et al. (2016).

In worker bees, HGs are paired structure located bilaterally in the head, in front of the brain between the compound eyes (Cruz-Landim and Costa, 1998; Hrassnigg and Crailsheim, 1998). Each gland is composed of thousands of two cell units, secretory cells, and duct cells (Cruz-Landim and Costa, 1998; Kheyri et al., 2012). These secretory cells (Figure 4) have a diameter of 30–50 μm and discharge their products into the highly convoluted canaliculus that wind around the nucleus and enclosed on its luminal side by a thin fenestrated cuticular lining known as the end apparatus (Kheyri et al., 2012; Richter et al., 2016). At one open end of the canaliculus, the secretory cell forms a thin tube-joint-like connection or ductule that is lined by a cuticular layer to the duct cell that is about 2 μm width and 50–100 μm length (Noirot and Quennedey, 1991; Kheyri et al., 2012). The place of attachment between the two cells can be depicted as a tube joint, where the secretory cell forms a sleeve of about 10 μm in length around the distal end of the duct cell (Richter et al., 2016). At the coupling position, the secretory and duct cells are connected by septate junctions and adherens junctions, the latter lying at the distal end of the canal cell and delimiting the open end of the canaliculus from the remaining area of the plasma membrane (Richter et al., 2016). Each acinus is determined by 6–20 two cell units; each cell is connected with a duct cell extending in a bundle to the collecting duct, around 60 μm in diameter (Kheyri et al., 2012). In each HG almost 800 acini are arranged around and along the collected duct that releases the secretion to the hypopharynx (Richter et al., 2016).

In newly emerged bees (NEBs), the HGs are in the phase of rapid development, small sized and well-structured cell cytoplasm with the acinus only being a cavity. Nuclei are larger and round with an obvious chromatin granulation and small vesicles around the nucleus (Škerl and Gregorc, 2015). They express a wide range of genes. The ribonucleoprotein complex biogenesis, fatty acid degradation, and highly abundant hexamerins (Hex 110) protein are all involved in regulating the young gland development and tissue construction in HGs (Boisvert et al., 2010; Karlas et al., 2010; Hu et al., 2019).

In nurse bees (NBs), secretory vesicles have developed on 3rd day; the acini size and number of secretory vesicles peak during 6–10 days (Deseyn and Billen, 2005). The intermediate filaments (actin filament) are important for the cytoskeletal component to maintain the appropriate shape of HGs which is necessary for their function (Kheyri et al., 2012). The up-regulated fibrous protein Lam (2.87-fold) in NBs may be critical for both structural and functional element of the cytoskeleton (Zhong et al., 2010; Kheyri et al., 2012; Goldmann, 2018). The β-actin and larger structures of the secretory cells of the acini in HGs of NBs are well-shaped as compared to those of NEBs and FBs (Feng et al., 2009; Kheyri et al., 2012; Goldmann, 2018). The morphological character of the secretory cell depends upon the secretory activity. The secretory activity is high in NBs. Thus, the secretory cells are large and the canaliculus between the actin rings is extended to the saccules (Kheyri et al., 2012), and the ovoid shape acini are linked via numbers of ductules to the collecting duct that extends along the total length of the HGs (Richter et al., 2016; Hu et al., 2019).

In forager bees (more than 15 days after emergence and having foraging duties), these glands are composed of several acini smaller than those of NBs. Moreover, the HGs gradually shrink and their secretory activity decreases (Ohashi et al., 1997) as a result of cell apoptosis (Britto et al., 2004) accompanied by decreased rough endoplasmic reticulum, smaller secretory cells with a thin canaliculus, and some saccules and suppressed protein synthesis rates (Knecht and Kaatz, 1990; Kheyri et al., 2012).

#### Development of Canalicular System and Actin Ring in Adult Worker Bees

The interesting characteristic of a canalicular system in the secretory cells of honey bee HGs is its actin rings that back the apical membrane tube at regular distance and that differ in various properties such as diameter, thickness, and F-actin content. Unique F-actin ring have a diameter of 2.5 μm and a width of 0.2–0.3 μm that links the plasma membrane of the secretory cell to the distal end of the duct cell (Kheyri et al., 2012; Richter et al., 2016). In newly ecdysed bees, some actin rings are connected to each other and are not completely closed. In NBs, the actin rings are of relatively uniform thickness and usually isolated from each other. Finally, in foragers, the actin rings are larger and varied in thickness (Richter et al., 2016).

## Factors Affecting the Development of HGs

The development of HGs is adversely affected by multiple biotic and abiotic stress factors (DeGrandi-Hoffman et al., 2010; Li et al., 2010; Al-Ghamdi et al., 2011; Wang et al., 2018; Ali et al., 2019; Corby-Harris et al., 2019). HGs of honey bee are age-dependent structures in which the size of the acini vary and are associated with different social behaviors (Ohashi et al., 1999; Deseyn and Billen, 2005; Richter et al., 2016), and the following factors.

### Honey Bee Races

**Figure 5:**
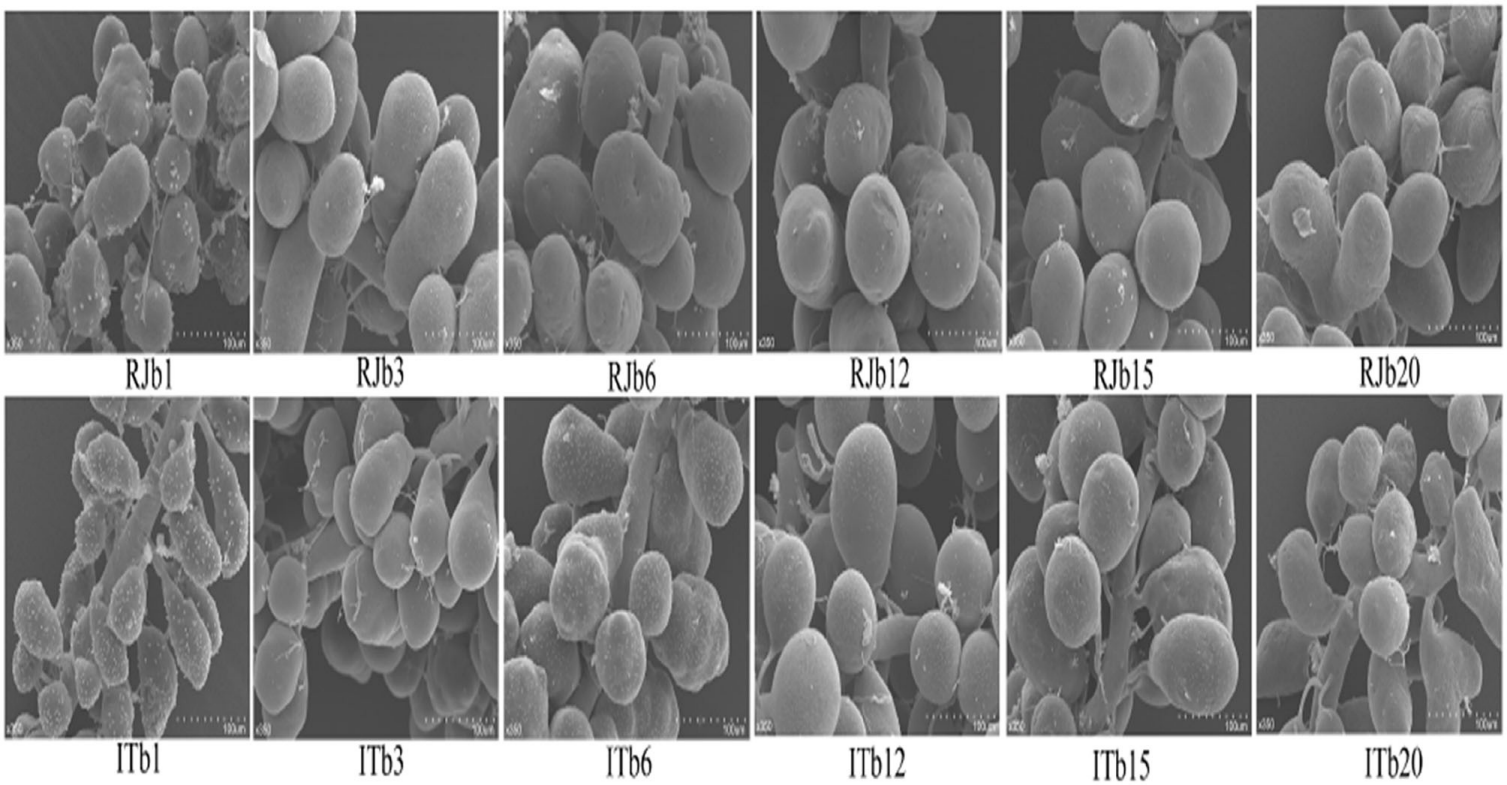
A represents of scanned electron microscope (SEM) profiles of hypopharyngeal glands (HGs) of royal jelly bees (RJBs) and Italian bees (ITBs) workers on day 1, 3, 6, 12, 15, and 20 at 350-fold magnification, respectively. Data used from Li et al. (2010).

The HGs are both studied in different honey bee races and within the same honey bee workers. At the pupal stage, the secretory cells are asymmetrical in shape with a small concentration of protein and carbohydrates, whereas secretory cells are well-developed in NBs, then the glands gradually decrease in size in the forager showing a smaller number of secretory vesicles (Kheyri et al., 2012; Škerl and Gregorc, 2015; Richter et al., 2016; Klose et al., 2017). The HGs of *A. florea* and *A. andreniformis* are very similar in terms of the histochemical structures, but differences are observed between pupae, nurses, and foraging bees. Pupae have an irregular and incomplete secretory unit with two to eight secretory cells. Nurse and foraging bees have completely developed secretory unit and composed of four to eight secretory cells with larger secretory vesicles (Suwannapong et al., 2007). Acini size (width and length) are larger in the HGs of NBs of *Apis mellifera* and *A. cerana*, then gradually decrease in size as the nurses become guards (Suwannapong et al., 2010). High royal jelly producing bees (RJBs, *A. mellifera ligustica*), a strain of honey bees selected from Italian bees (ITBs), possess larger acini size as compared to ITBs (Li et al., 2010; Hu et al., 2019). The diameter of acini (Figure 5) in both stocks increases in a similar manner until 12 days after which they get smaller (Li et al., 2010).

### Food Resources

Food availability affects the development of HGs in honey bees. Pollen protein contents and its quality affect the performance and lifespan of honey bee colonies. In contrast, poor pollen quality leads to weight loss, less protein content, reduced longevity, and partial development of HGs in honey bees (Schmidt et al., 1995; Brodschneider and Crailsheim, 2010; Nicolson, 2011). Bees that feed on naturally monofloral pollen from *Castanea* sp. or *Asparagus* sp. or pollen mixtures have larger HGs as compared to those that feed on pollen from *Helianthus* sp. and *Sinapis* sp. (Omar et al., 2017). Pollen diet, brood pheromone, and queen mandibular pheromone plus brood pheromone treatment significantly enhance the protein concentration in the acini of HGs (Peters et al., 2010). In contrast, worker bees consume more pollen than protein supplement (MegaBee), but there was no effect of either diet on the protein contents and size of acini in HGs (DeGrandi-Hoffman et al., 2010). Similarly, the development of the HGs of young worker bees is not affected by ingestion of (cotton cultivar) CCR141 pollen in control environments (Han et al., 2012). The greatest acinal area of HGs was observed when honey bees fed on bee breed followed by pollen load and a mixture of yeast, gluten, and sugar (1:1:2) (Al-Ghamdi et al., 2011). Honey bees fed on the first 3 days on sucrose solution had a lower level of proteolytic activity and decreased size of HGs compared to those fed on honey and beebread (Crailsheim and Stolberg, 1989). L-proline syrup (1,000 ppm) has a positive impact on the development of the HGs and could be considered as a phagostimulant role of syrup consumption in the honey bees (Darvishzadeh, 2015). Also, a larger acinal area of HGs was present when worker bees feed on 100 and 200 ppm of thiamine (Vitamin B1) at 3rd and 6th days of the age (Mohebodini et al., 2013). Bees fed with vitamin C had a wider HGs acini size followed by soya bean flour and skim milk (Zahra and Talal, 2008).

### Seasonal Factors

The cells of the HGs of winter bees contain a large number of secretory vesicles. The secretions are stored until spring and reactivated as the workers use them to feed a new generation of larvae (Deseyn and Billen, 2005). The NBs have larger acini size as compared to the foragers of all races (*A. mellifera jemenitica* Ruttner 1976, *A. mellifera carnica* Pollmann 1879, and *A. mellifera ligustica* Spinola 1806) during winter and summer seasons. The foragers in all bee races presented higher lipofuscin accumulation than nurse bees during both seasons. Thus, irrespective of seasons, the acini size and lipofuscin accumulation are inversely correlated with each other in the nurses and foragers of all bee races (Ali et al., 2019). However, acini size of *A. mellifera* and *A. cerana* are larger during summer as compared to winter season (Shakeel et al., 2020). The acinar cells of spring worker bees have more developed organelles, such as secretory granules and endoplasmic reticulum indicating vigorous secretory activity of cells as compared to overwintering bees (Wang et al., 2018).

### Pesticide Exposure and Other Factors

Pesticide exposure could negatively affect brood development, survival, and colony maintenance. Pesticides cause apoptotic and necrotic cell death in the HGs (Škerl and Gregorc, 2010). Six days of pyraclostrobin and fipronil treatment did not affect the HGs, but these pesticides reduced the larger acini size and thus caused decreased RJ secretion by the NBs (Zaluski et al., 2017). The cytotoxic effect of the herbicide alters the cellular ultrastructure of the HGs causing premature degeneration of endoplasmic reticulum and morphological and structural variations in mitochondria that could reduce their bioenergetic functions such as ATP production and lead to apoptosis (Faita et al., 2018). In the HGs of infected and fumagillin treated bees, a crystalline structure is seem in some of the secretion granules, which may exhibit a low rate of protein synthesis (Liu, 1990). The fenoxycarb pesticide has an adverse effect on HGs size and secretory activity while the effects of captan, imidacloprid, and indoxacarb treatments are not clear yet (Heylen et al., 2011). In contrast, a sublethal dose of imidacloprid negatively affects the development of HGs in laboratory conditions (Hatjina et al., 2013). Pollen diets containing 1% of soybean trypsin inhibitor (SBTI) also had an adverse effect on the development of HGs, midgut protease activities and the existence of bees (Sagili et al., 2005). However, transgenic products such as aprotinin, avidin or Cry1Ba protein, and CCRI41 had no influence on the development of HGs (Malone et al., 2004; Han et al., 2012).

Nutrient stress causes lipolysis in the fat body, which may release the sterols into the hemolymph that are vital for ecdysteroid synthesis; and enhanced ecdysteroid levels caused autophagic degradation and reduced the function of HG of starved bees (Corby-Harris et al., 2019). *Varroa* mite infestation reduces the activity of HGs by decreasing the acinal surface area, the number of secretory vacuoles and the quantity of secretion in gland duct (Yousef et al., 2014; Ayoub et al., 2015). The injection of juvenile hormone into the honey bee activates the lysozyme that leads to the degeneration of the HGs (Liu, 1989). Bee venom harvesting negatively affects the structure of HGs and resulted in a decrease in the production of RJ (Bovi et al., 2017).

## Molecular Insight into the Development and Physiology of HGs

Advancement in honey bee genome sequence and “omics” techniques significantly extend our understanding the biology, behavior, physiology, neurobiology, and immunology of honey bees at molecular and biochemical levels (Hora et al., 2018). Molecular comparison of honey bee worker HGs is critical for the mechanistic understanding of its development and functionality. Hence, the HGs express unique genes in the NBs and FBs to fit their age-related tasks. Specifically, genes of major royal jelly proteins (*mrjps*) and ribosomal proteins are expressed higher in NBs, whereas they rapidly decrease their expression in foragers (Ohashi et al., 1996; Ueno et al., 2009; Liu et al., 2014, 2015). In foragers, genes of α-glucosidase, glucose oxidase, galactosidase, lipase, amylase, esterase, invertase, and leucine arilamidase are consistent expressed in HGs and responsible to the processing of nectar into honey (Kubo et al., 1996; Ohashi et al., 1996, 1999; Deseyn and Billen, 2005).

**Figure 6:**
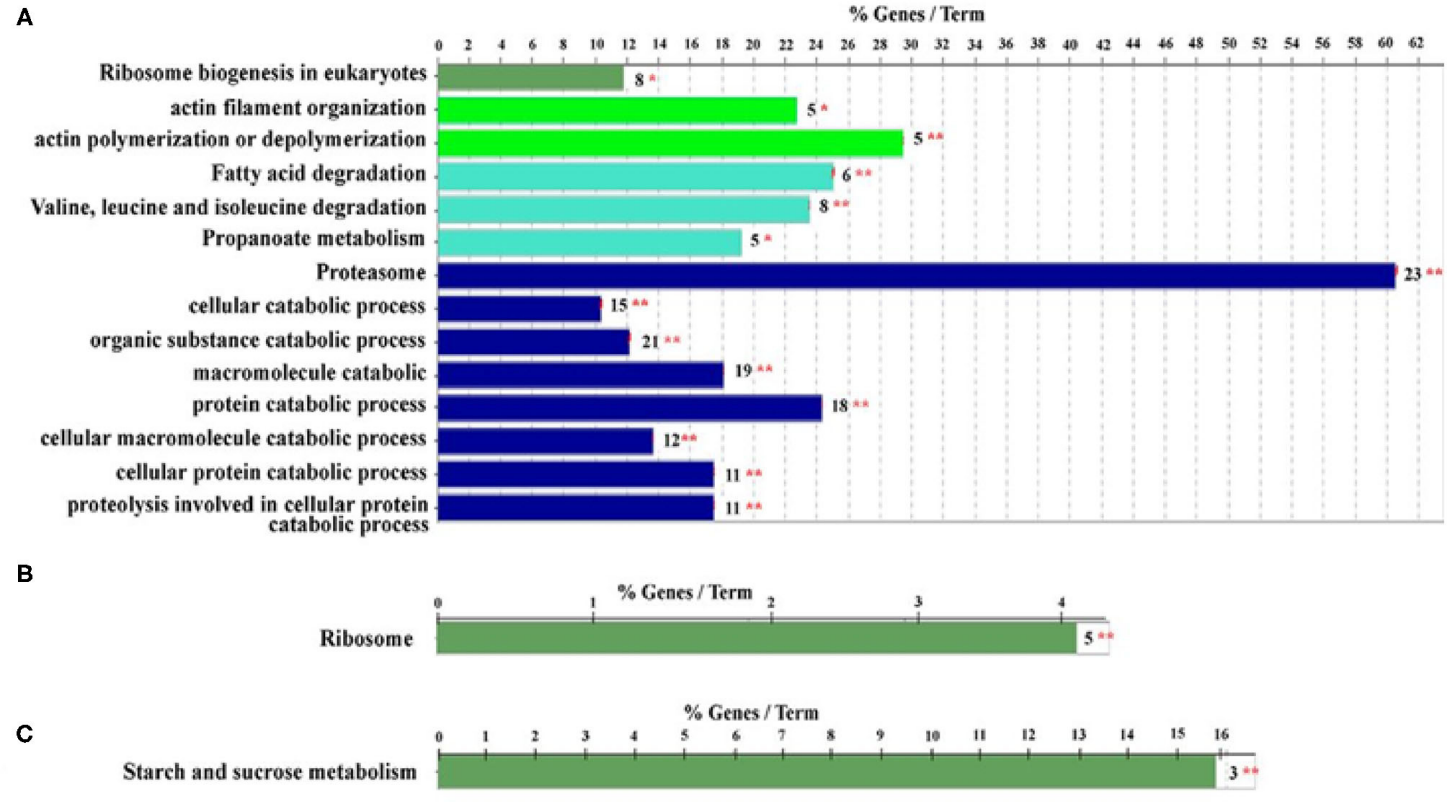
Qualitative proteome comparison of age-specific hypopharyngeal glands (HGs) in royal jelly bees (RJBs). **(A–C)**, the functional classes enriched by upregulated proteins (fold change ≥ and *p* < 0.05) in newly emerged bees (NEBs), nurse bees (NBs), and forager bees (FBs), respectively. % genes/Term stands for the proportion of genes enriched in the corresponding functional groups. The bars with the same color represent the same functional groups. The number stands for the genes enriched to the corresponding functional groups and asterisks sign represent *p < 0.05; **p < 0.01. Data used from Hu et al. (2019).

Furthermore, proteomics has also become a powerful tool for revealing the HGs morphology and physiology that supports honey bee biology (Altaye et al., 2019b; Hu et al., 2019). Notably, the potential of secreting RJ in the HGs of ITBs and RJBs is varied. In RJBs, the HGs develop the potential to secrete RJ on day 3 after emergence as seen by the identification of MRJPs in the HGs, whereas ITBs do not secrete RJ until day 6 after emergence (Feng et al., 2009), and their peak level of RJ secretion occurs within 6–12 days at the nurse stage. Moreover, comparison of protein expression difference in HGs at a specific phase of physiology between ITB and RJB workers reveal that RJB workers modify a proteome strategy to enhance RJ production by increasing the wide range of proteins as compared to ITB workers. For instance, the HGs of RJBs considerably up-regulate a wide range of pathways engaged in carbohydrate metabolism, energy production, protein biosynthesis, antioxidants, development, transporters, and cytoskeleton, indicating their functions to assist the development and secretory activities (Li et al., 2010). Recently, the induced activity of actin polymerization or depolymerization, isoleucine and valine degradation, proteasome, and ribosome biogenesis in NEBs are to support the structural ontogeny of HGs (Figure 6A). The ribosome protein synthesis and energy metabolism are important for the production of RJ in NBs (Figure 6B), while sucrose and starch metabolism are functionally induced at the forager stage (Figure 6C). Furthermore, the strong expression of proteins related to protein biosynthesis and energy metabolism, such as RpS27, RpL13, RpL4, RpS4, MDH, GltA, Hex 110 in the HGs of RJB nurses provides solid evidence to enhance RJ production (Hu et al., 2019). Moreover, the phosphoproteome of the HGs at each worker stage demonstrates that a large portion of cellular protein are phosphorylated and could play a vital role in HG activity (Qi et al., 2015). The phosphorylation modifications of the ribosomal proteins of HGs may be vital for the high efficiency of synthesizing and secretion of RJ protein (Lu et al., 2013). The protein abundance levels and phosphorylation events are not associated, and most of HGs proteins are controlled by phosphorylation independently of their expression level in the honey bees. The functional classes such as pyruvate metabolic process, muscle cell development, and pole plasma mRNA localization are functionally complementary in NEBs and NBs of the proteome and the phosphoproteome while pyruvate metabolic process and muscle cell development classes are enriched in foragers in a non-phosphoproteome (Qi et al., 2015). Such evidence indicates that both phosphoproteome and non-phosphoproteome are essential to perform complementary biological functions to support the unique age-dependent physiology of the HGs. In short, protein phosphorylation is independent of its expression, and complementary protein and phosphoprotein expression profiles are essential for the distinct physiology of secretory activity in the HGs (Qi et al., 2015).

A multifaceted network of molecular pathways supports the development and functionality of HGs in honey bees. Pathways of insulin-like growth factor (IGF), the mammalian target of rapamycin (mTOR), and transforming growth factor-beta (TGF-β) signaling involve nutrient availability, intrinsic developmental program, metabolism (Barchuk et al., 2007; Patel et al., 2007; Alaux et al., 2011), life span, and reproduction (Colombani et al., 2003; Oldham and Hafen, 2003), and cell differentiation, cell death, and motility (Ikushima and Miyazono, 2011). They play a dominant role in the development and physiology of the HGs, which lead to RJ and enzyme secretion (Ohashi et al., 1997, 1999; Chanchao et al., 2006). Moreover, the ribosomal pathway is central for the secretion of RJ proteins in the HGs and it is regulated by phosphorylation events reflected as an age-specific expression of various ribosomal units (Qi et al., 2015). The Warts (wts) is a signaling pathway that control organ size through the regulation of cell proliferation and apoptosis (Lai et al., 2005; Lee and Tournier, 2011). The wts pathway observed in NBs and FBs suggest that phosphorylation is key to the wts pathway (serine/threonine-protein kinase ULK 12) that regulates autophagy (Lee and Tournier, 2011), cell survival, homeostasis, and promotes development in living cell (Levine and Klionsky, 2004; Komatsu et al., 2005) for the normal function of the HGs at different ages. However, the distinct role of identified proteins and phosphoproteins in associated specific biochemical pathways concerned in the development and functionality of HGs needs to be further investigated.

## Conclusions and Future Research Direction

Understanding the morphogenesis of honey bee HGs and the factors which regulate HG development is essential for further investigations of the functionality of the glands. HGs are age-dependent structures in honey bees that change with the acinus size and correlate with different social behavior. Various factors including honey bee races, food, colony size, season, pesticides, and Varroa infestation affect the ontogeny and physiology of HGs. In the pupal stage, the HGs are comprised of incomplete structures and irregular shape of secretory units. In worker honey bees, each gland consists of thousands of two cell units, secretory cell, and duct cell. HGs of NBs are well-developed and synthesize RJ; while in FBs, the glands shrink and synthesize α-glucosidase, glucose oxidase, and invertase that convert nectar into honey. In addition, a subset of genes, proteins, and phosphoproteins underpin the morphogenesis and physiology of HGs. Further studies using gene-editing tools are required to investigate the specific function of genes or protein that regulate the ontology and physiology of HGs in the worker bees.

## Author Contributions

SA prepared the original draft. SK and KK review the manuscript. JL conceived the manuscript and supervision. All authors have read and agreed to the published version of the manuscript.

## Conflict of Interest

The authors declare that the research was conducted in the absence of any commercial or financial relationships that could be construed as a potential conflict of interest.

## Abbreviations

ATP: Adenosine triphosphate
ER: Endoplasmic reticulum
FBs: Forager bees
HGs: Hypopharyngeal glands
IGF: Insulin-like growth factor
ITBs: Italian bees
MRJPs: Major royal jelly protein
NEBs: Newly emerge bees
NBs: Nurse bees
PTMs: Post-translation modifications
RJBs: Royal jelly bees
RJ: Royal jelly
TCA: Tricarboxylic acid
TGF-β: Transforming growth factor beta
TOR: Target of rapamycin.
